# The role of whistleblowers in protecting the safety and integrity of the food supply

**DOI:** 10.1038/s41538-018-0017-5

**Published:** 2018-05-08

**Authors:** Gerald G. Moy

**Affiliations:** Food Safety Consultants International, 6 Chemin des Peutets, 1253 Vandoeuvres, Switzerland

**Keywords:** Agriculture, Economics, Science, technology and society

## Abstract

The unsafe, illegal, and unethical practices, especially food fraud, by various actors along the food supply chain are old problems that have been made even more challenging by today’s international agri-food network. To combat these practices, the internal reporting to upper management or the external disclosure to regulators or media by persons who become aware of such practices is essential. These persons are colloquially known as whistleblowers. However, a number of impediments to whistleblowing, especially retribution by employers, have limited their contribution to ensuring the safety and integrity of the food supply. This paper presents recent examples of whistleblowing and legislation adopted by countries around the world to encourage and protect whistleblowers, especially from retaliation. Several impediments to whistleblowing are described and suggestions for overcoming them are proposed.

## Introduction

From the earliest civilizations, those seeking economic gain have at times subjected the food supply to unsafe, illegal, and unethical practices. While only a small number are thought to be involved, perpetrators of such practices can include anyone who has access to the food supply chain from producers to retailers. The current size and complexity of the international agri-food network makes the food supply chain more vulnerable to such unscrupulous practices. In some cases, organized crime is involved in a food business as part of a larger criminal enterprise. If discovered, companies that undertake such practices may face expensive recalls, criminal penalties, liabilities, and possible bankruptcy. Such practices can also result in significant economic losses for the food industry as a whole. Ethical companies may lose market share to unethical competitors who are able to sell their products at lower prices. If a particular food is implicated as unsafe or fraudulent, consumers may lose confidence in the safety of all brands of that food. This can cause losses for food businesses that are honest and law abiding. Other losses are the general erosion of confidence in the safety and integrity of the food supply and in good governance by responsible authorities. While food fraud harms the consumer economically, potential risks to health and nutrition are major concerns.

In Medieval Europe, trade guilds established ethical codes maintained largely by peer pressure to address this problem. In modern times, the food industry has continued to promote the safety and integrity of the food supply through cooperative programs. For example, the Global Food Safety Initiative (GFSI) is a collaborative effort among the world’s leading food safety experts from manufacturing, retail, and food service companies to provide continuous improvement in food safety management systems to ensure confidence in the delivery of safe food to consumers. The GFSI benchmarks food safety standards for manufacturers as well as safety assurance standards for farms.^1^

While the food industry has primary responsibility for the safety and integrity of the foods they produce, countries have enacted laws governing food to promote a safe and honestly presented food supply. Enforcement of these laws is now universally considered to be an essential public health function of any government. The media also plays a critical role by informing the public of unsafe and fraudulent food products. They also help to mobilized consumer demand for better food safety protections. In the early part of the last century, the first food safety law in the United States was precipitated by an investigative journalist who exposed the unsanitary conditions in the Chicago stockyards.^2^

**Table 1:** Whistleblower legislation in selected countries

Country	Legislation	Sector	Coverage	Comment
Australia	Public Interest Disclosure Act 2013	Public	Government corruption and wrongdoing	Commonwealth Ombudsman for food issues
Canada	Public Servants Disclosure Protection Act 2007	Public	Government wrongdoing	Journalistic Source Protection Act was adopted in 2017
China	Whistleblower Regulations 2016	Public	Government corruption and wrongdoing	
European Union	Council of Europe Recommendations 2014	Public/Private	Serious threat or actual harm to the public	Principles to be adopted by member countries
India	Whistleblower Act 2014	Public	Government corruption	
Japan	Whistleblower Protection Act 2004	Public/Private	Violation of laws and regulations	
Republic of Korea	Protection of Public Interest Whistleblowers Act 2011	Public/Private	Violation of law, harm to health, safety or environment, consumer interest, fair competition	Strengthen by amendment in 2017; includes monetary incentives
New Zealand	Protected Disclosure Act 1999	Public/ private	Unlawful, corrupt or irregular acts, serious risk to health, safety, or environment	
South Africa	Protected Disclosures Act 2000	Public/Private	Breach of law, miscarriage of justice, danger to health and safety, damage to environment, unfair discrimination	
United Kingdom	Public Interest Disclosure Act 1998	Public/Private/food industry	Breach of law, miscarriage of justice, danger to health and safety, damage to environment	National Food Crime Unit established by Food Standards Agency
USA	Food Safety Modernization Act 2011	Food industry	Any violation of Food, Drug, and Cosmetic Act	Includes monetary incentives under other legislation
Vietnam	Law on Denunciation No. 03/2011/QH13 2011	Public/Private	Criminal violation or corrupt act	Failure to denounce may be subject to penalty

## Challenges of the globalized food supply chain

Today the food sector is one of the largest and most important areas of economic productivity in almost every country. In 2016, the world food and agriculture market was worth about US$ 8 trillion^3^ and in 2015 global trade in food exceeded US$ 1.3 trillion.^4^ Within this complex international network, food businesses must rely on all parties meeting their responsibilities to provide safe and honestly presented products. However, long food supply chains that cross multiple jurisdictions are vulnerable to fraud, for example, the horsemeat scandal in Europe. While food fraud is mostly an economic issue, the temptation to illegally cut corners can inadvertently turn into a food safety crisis. The 2008 melamine incident in China is a case in point where melamine was added to watered-down raw milk to fool quality tests. When children and infants consumed the adulterated milk and infant formula made from the milk, about 300,000 of them experienced adverse kidney and urinary tract effects, such as kidney stones, including six reported deaths. This was one of the biggest food fraud cases to every be uncovered in which tainted products were exported to over 40 countries.^5^

The importance of whistleblowers in protecting the safety and integrity of the food supply is illustrated by a major salmonella outbreak that occurred in the USA in 2008. Kenneth Kendrick was the assistance plant manager at a Texas peanut processing plant when he observed serious hygienic problems and improper quality control practices that were being perpetrated by the management of the now-defunct Peanut Corporation of America. Well before the outbreak occurred, he attempted to disclose this to state government and the food industry, but no one appeared interested. When the outbreak of salmonellosis caused by contaminated peanuts finally occurred, thousands of people were made ill, including nine reported fatalities. As the peanuts were used extensively in a variety of finished products, a massive recall ensued with the total cost to the food industry estimated to be over US$1 billion. The company went out of business and several company officials were sentence to prison for their actions.^6^ In response to this and other incidents, whistleblower protections in the USA were incorporated into the Food Safety Modernization Act (FSMA) of 2011.

A whistleblower’s perspective is unique in that she or he is on the job every day and is often more knowledgeable about an operation and a product than anyone else. Responsible management needs internal whistleblowers to provide critical information from the “shop floor” to prevent costly mistakes that can lead to severe financial loss and harm to the public. Management should proactively seek to address safety problems as part of its public health posture, which is required by most modern food safety legislation. This also contributes to food defense, which is growing concern in some countries. Moreover, a robust internal whistleblowing system is simply a prudent business practice that facilitates communication. For example, an effective internal whistleblowing system between engineers and management could have prevented the Challenger disaster.^7^

Considering the size and complexity of the international agri-food network, the industry has done a remarkable job in assuring the safety and integrity of the food supply. In this regard, industry controls are far more important than the limited controls of the governments. Often government resources can only allow inspections of food premises to be conducted once per year. Even a well-funded regulatory body, such as the US Food and Drug Administration, samples <1% of all of the food imported into that country.^8^ Under these conditions, unsafe and fraudulent foods, such as the melamine contamination of milk in China and the horsemeat scandal in Europe, are difficult to detect and remedy. With proper protections and incentives, whistleblowers who are in a position to observe such practices first-hand, could alert upper management or outside officials to such practices. Whistleblowers should, therefore, be considered as essential players in any effective system to ensure food safety and integrity and should be encouraged and protected from retribution.

## Whistleblowing legislation around the world

One definition of a whistleblower is provided by the European Council, namely: “Any person who reports or discloses information on a threat or harm to the public interest in the context of their work-based relationship whether public or private”. In this context “reports” refers to an internal a whistleblower who exposes a misdeed within a company or organization. In comparison, “discloses” refers to a whistleblower who shares information about a misdeed to a government authority or the public, including the media. Usually, a whistleblower will first seek an internal resolution to a problem. If the channel for such reports does not exist or does function properly, a whistleblower may decide to go to the outside to seek redress. In general, members of the public, including journalists, are not referred to as whistleblowers.

Because whistleblowers may be at risk of retribution, certain protections have been afforded to them. However, in many cases, these protections have only recently been granted and much of the legislation is intended to encourage workers in the public sector to report on misdeeds or corruption. However, to protect public health and safety, most developed countries have adopted legislation to cover both the public and privates sectors. In the recognition of the importance of food safety and public health, a few countries have adopted specific legislation for employees in the food sector. The following is a sampling of general and specific legislation in various parts of the world to encourage whistleblowers and protect them from retribution. An effort has been to update this information through the end of 2017.

### North America

In the United States, the 2011 FSMA offers comprehensive protections to whistleblowers who have provided information relating to any violation of the food safety and fraud regulations of the US Food, Drug and Cosmetic Act to their employer, the Federal Government, or the attorney general of a state.^9^ Also covered are whistleblowers who have testified, assisted, or participated in a proceeding concerning a violation of FSMA and its regulations; and those that objected to or refused to participate in any activity that he or she reasonably believed to be in violation of the food regulations. Retaliation against an employee for whistleblowing is specifically prohibited, including protection against firing or reassigning, reducing pay or overtime, blacklisting or failing to hire, demoting or denying promotions, denying benefits, intimidating or making threats, and disciplining. In addition, a law dating from the US Civil War in the 1860s provides that whistleblowers, including those in the food sector, who reveal that false claims or fraud were made against the Federal Government, are entitled to monetary rewards.^10^ From 2011 to the end of 2016, 280 whistleblower complaints have been filed under FSMA. Of these, 62 cases were resolved to the satisfaction of the employee and another 50 cases are still under investigation. Two other cases resulted in prosecutions. About 165 other case were withdrawn or dismissed, mainly because the whistleblower had failed to provide addition information.^11^

Canada, in part spurred by the recall of contaminated raw chicken products, is considering strengthening its whistleblower protections that were first adopted in 2007 as the Public Servants Disclosure Protection Act. Among the recommendations being considered is that the onus of responsibility for showing retaliation or the lack thereof would be shifted from the whistleblower onto the employer. Also, investigation of possible wrongdoing would be extended to the private sector from just the public sector. Before the end of 2018, the government is scheduled to consider these changes.^12^ However, the Journalistic Source Protection Act was adopted 2017, which provides anonymity to whistleblowers in the public and private sectors.^13^

### South America

In 2010, Peru adopted a whistleblower law to protect public sector employees. Other countries, including Mexico, Guatemala, and Columbia, are considering whistleblower legislation.

### Europe

Within the European Community (EC), the Council of Europe has developed a legal instrument to protect individuals who report or disclose information on misdeeds that pose a serious threat or actual harm to the public. In 2014, the Committee of Ministers adopted a recommendation on the protection of whistleblowers that sets out a series of principles to guide member countries in developing national legislation. These were based on a document prepared by the Organization for Economic Cooperation and Development (OECD) at the request of the G20 Leaders when they met in November 2010. Many EC and other European countries have developed legislation consistent with these principles.^14^

Since 1998, the United Kingdom has implemented the Public Interest Disclosure Act that protects whistleblowers from retaliation if they expose wrongdoing.^15^ The UK Food Standards Agency has applied these protections to food industry personnel who disclose activities that constitute a criminal offense, a breach of a legal obligation, a miscarriage of justice, a danger to the health and safety of any individual, damage to the environment, and a deliberate concealment of the five fore mentioned matters. The Food Standards Agency has also established the National Food Crime Unit (NFCU) to protect consumers from serious criminal activities that can affect the safety or authenticity of food. The NFCU has encouraged food businesses, whistleblowers, and consumers to report suspicious food or questionable activities on a confidential basis via a telephone or internet hotline. This includes matters where food has potentially been adulterated or substituted, methods used in workplaces for producing, processing, storing, labeling, or transporting food that appear to be unsafe or illegal, and companies selling substandard food that is purported to be of a certain quality or nature, suggests health benefits or claims to be from a specific place or region, but do not appear genuine or are suspected to be fake.^16^ A total of about 70 whistleblower complaints have been filed with the NFCU through the end of 2017. The NFCU could not confirm or deny that a whistleblower was responsible for alerting authorities to the horsemeat scandal (personal communication with the UK National Food Crime Unit (11 January 2018)).

However, in September 2017, a whistleblower who had been a government inspector exposed the purported unhygienic and poor animal welfare conditions at several slaughterhouses in the UK. While stressing that no meat is completely sterile, food safety experts say that many of the unhygienic practices revealed by the whistleblower and confirmed by official reports increased the likelihood of contaminated meat reaching the public, thus increasing the chances of serious foodborne disease outbreaks. The whistleblower also revealed the intimidation of inspectors and veterinarians by abattoir operators that impeded their public health work.^17^

In Russia, no specific legal protection for whistleblowers exists. A 2004 law offers government protection for victims, witnesses, and other participants in criminal court proceedings and technically this would cover whistleblowers. In order to be eligible for protection, however, individuals must go public with their information and participate in court proceedings, which may be a long and difficult process.^18^

### Asia and Pacific

In March 2016, the Chinese Ministry of Public Security, Ministry of Finance and Supreme People’s Procuratorate released several regulations protecting and rewarding whistleblowers. The regulations are intended to strengthen the government’s efforts to eliminate corruption by government officials. These protections are extended to the whistleblowers and their close relatives and include protections for their property, employment and job status. Local police play an important role in protecting whistleblowers and their close relatives.^19^ In addition, existing labor laws specifically prohibit retaliation against whistleblowers. China has set-up an official website for whistleblowers to report government corruption and other wrongdoings. To encourage whistleblowing, a mobile phone application was developed in 2015 and a social media account was established in 2016.

Australia’s first national law providing legal protection for government whistleblowers, the Public Interest Disclosure Act, was adopted 2013 and took effect at the beginning of 2014. The Commonwealth Ombudsman oversees the whistleblowing framework related to the food industry. In New Zealand, the Protected Disclosure Act applies to public and private sector employees and came into force in 2000. In Japan, the Whistleblower Protection Act was enacted in 2004 to protect public and private sector employees and came into force in 2006. In 2011, the Republic of Korea adopted the Protection of Public Interest Whistleblowers Act to provide legal protections for public and private sector employees. The law provides monetary rewards of up to US$ 2 million if a report leads to savings by government agencies. In Vietnam, the government issued a decree in 2012 to protect all Vietnamese citizens and foreign individuals from a wide range of retaliation, including threats to their life, health, property, honor, dignity, and position, as well as to those of their relatives.^20^ In 2014, India enacted the Whistleblowers Protection Act intended to protect whistleblowers in the public sector, but this may include persons outside government and non-governmental organizations. Prosecution of corruption has mainly proven effective among the lower levels of India’s bureaucracy and the government has not fully operationalized the act. Proposed amendments to the act have proven controversial.^21^

### Africa

Across nations of Africa, corruption in many countries has been a major problem leading to economic inequality and developmental stagnation. The inability to expose wrongdoing without fear of consequences is widespread. However, a number of countries have enacted legislation to protect whistleblowers, including Cote d’Ivoire (2009), Ghana (2006), Morocco (2011), Mozambique (2011), and Zambia (2010) In 2017, the Nigerian government adopted legislation to protect whistleblowers from retaliation, including allowing anonymous reporting. In addition, governments in Angola, Botswana, Kenya, and Tunisia are considering legal protections for whistleblowers.^22^

In South Africa, the Protected Disclosures Act was adopted shortly after the end of Apartheid in 2000. However, research in 2014 showed that only 3 in 10 South Africans felt safe blowing the whistle, demonstrating that good laws are only one part of the solution to the problem of entrenched corruption. A summary of whistleblower legislation in selected countries is given in Table 1.^22^

## Impediments to whistleblowing

The first impediment may be cultural because in many countries, some people associate whistleblowers with certain negative stereotypes, such as those who ingratiate themselves with authority figures. Some persons may also believe that whistleblowers are disgruntle employees with psychological problems. Others may view whistleblower as having illegally obtained their information, such as hackers who have stolen information through the internet. At times, information may relate to national security, in which case whistleblowers may be cast as disloyal. In some cases, whistleblowers have been motivated by monetary rewards. Whistleblowers might also make their colleagues uncomfortable because many of them have stayed silent although they were aware of the wrongdoing. This all may lead to the isolation and shunning of whistleblowers, which serves to dissuade other potential whistleblowers. In reality, whistleblowers often pay a high price for their reporting or disclosures.^6,23^

A second impediment is the low socio-economic status of many food industry workers that makes them vulnerable to retaliation. Many are minimum-wage employees that may have little incentive to report illegal or unethical practices that they might observe. Often they may not be aware of company policies and mechanisms for internal reporting and of the legal protections that are in place to prevent retribution. The fact that some of the biggest food fraud scandals were not reported by whistleblowers indicates that whistleblowing, while good in concept, may not be wholly reliable in practice.

The third impediment is that internal reporting systems of many companies may be flawed or even non-existent. Some companies may have even suppressed the whistleblower’s information and have subjected whistleblower to retaliation. In countries with no legal protections, a whistleblower may be forced to deal with a company which often have significant legal resources. Other businesses may have established internal whistleblowing policies and procedures, but their implementation may have been ad hoc and subject to conflicts of interest. For example, a whistleblower may be required to report to her or his immediate supervisor who may be the cause of the problem in the first place. Finally, some managers may operate in the negative mode—that is, they use punishment instead of reward to motivate their employees. Under such conditions, personnel may be discouraged from internal whistleblowing.^23^

The fourth impediment resides in governments. While some progress is being made, many countries do not have any protections for whistleblowers and most do not specifically address whistleblowers in the private sector. In a critical industry such as food, this may lead managers to limit their internal whistleblowing efforts. Another factor is that government enforcement priorities are usually based on health significance and few resources are available for consumer fraud issues. Consequently, some whistleblower tips involving fraud may not be pursued. If nothing is done with the information provided, potential whistleblowers may become reluctant to inform outside authorities of problems. Even when legislation exists, some governments may not provide effective protection for qualified whistleblowers from retaliation by their employers. Some governments have even prosecuted whistleblowers for disclosing protected or confidential information in spite of the illegal activities that were revealed.

## Suggestions for improvement

To ensure the safety and integrity of our food supply, efforts should be made to overcome some of the impediments to whistleblowing. The role of whistleblowing as part of an effective food crime control system, especially for small- and medium-sized organizations, has been extensively reviewed.^24^ The following general suggestions are offered, but further research is required to specifically identify critical factors and how they should be addressed. However, the need for whistleblowing legislation to protect persons working in the food industry should be considered a priority.

Cultural perceptions should be improved by recognizing whistleblowers for their civic responsibility, professionalism, and personal courage. In particular, whistleblowers should be seen for their positive contributions to the safety and integrity of the food supply with benefits for the food industry, the government and particularly, the health and welfare of consumers. Efforts should be made to improve the socio-economic status of food industry workers and to encourage their awareness of unsafe, illegal, and unethical practices.

In regard to the food industry, employees should be encouraged to report any behavior or suspicious activity that may be unsafe, illegal, or unethical. This is particularly important with regard to Critical Control Points, which may impact on food safety. To facilitate reporting by employees, food companies should establish policies and procedures for effective and efficient internal whistleblowing with channels to top management. All employees should be informed of internal reporting procedures and their rights and protections as whistleblowers.

Most importantly, governments should adopt legislation to protect whistleblowers in the public and private sectors from retaliation, particularly those working in the food industry because of its importance to health and safety. Considering the size and complexity of today’s globalized food supply network, governments should recognize whistleblowing as an essential complement to their efforts in keeping unsafe, illegal, and unethical practices in check.

## References

[1] Global Food Safety Initiative. www.mygfsi.com (2018).

[2] Sinclair, U. *The Jungle*. (Doubleday, Jabber & Company, New York, NY, 1906).

[3] Plunkett Research, Ltd. *Plunkett’s Food Industry Market Research*, www.plunkettresearch.com (2016).

[4] World Trade Organization. *World Trade Statistic Review 2016* (Geneva, Switzerland, 2017).

[5] Gossner CME (2009). The melamine incident: implications for international food and feed safety. Environ. Health Perspect..

[6] Detwiler, D. Blowing the whistle on wrongdoings. *Food Quality and Safety*, www.foodqualityandsafety.com (2015).

[7] Rogers Commission. *Report of the Presidential Commission on the Space Shuttle Challenger Accident* (Washington, DC 1986).

[8] US Government Accounting Office, Imported Food Safety: FDA’s Targeting Tool Has Enhanced Screening But Further Improvements Are Possible, GAO-16-399 (Washington, DC 2016).

[9] US Food and Drug Administration, *Food Safety Modernization Act* (Washington, DC 2011).

[10] US Federal Government, *False Claims Act of 1863* (Washington, DC 1986).

[11] Flynn, D. FSMA whistleblower clause protects those who report problems. *Food Safety News* (2017).

[12] Ireton, J. Report calls for revamping of whistleblower law. (CBC News Ottawa, Canada, 2017).

[13] Canadian Government. *The Journalistic Source Protection Act of 2017*. Available at: https://news.vice.com/en_ca/article/3kp7n3/canada-passes-law-to-protect-whistleblowers-and-journalists-confidential-sources (2018).

[14] Council of Europe. *Recommendation CM/Rec 7 of the Committee of Ministers to Member States on the Protection of Whistleblowers*. Brussels. Available at: https://rm.coe.int (2014).

[15] United Kingdom Government. *Public Interest Disclosures Act 1998*. www.legislation.gov.uk/ukpga/1998/23contents (1998).

[16] UK Food Standards Agency. *Whistleblowing*, www.food.gov.uk/enforcement/the-national-food-crime-unit/foodfraud/whistleblowing (2017).

[17] Davies M. & Wasley A. Blowing the whistle on the meat industry: An insider’s account of work pressures in the slaughterhouse. *The Bureau of Investigative Journalism*, www.thebureauinvestigates.com/stories/2017-09-19/blowing-the-whistle-on-the-meat-industry (2017).

[18] Russia – Whistleblowing Protection. *Blueprint for Free Speech*, https://blueprintforfreespeech.net/document/russia/ (2013).

[19] vandePol, M., Wu, V. & Hui, S. New rules offer greater protection and incentives to whistleblowers in China. *Global Compliance News*, https://globalcompliancenews/new-rules-offer-greater-protection-and-incentives-to-whistleblowers-in-china-20160504/amp/ (2016).

[20] Whistleblower Protection Laws. *Blueprint for Free Speech*, https://blueprintforfreespeech.net/whistleblowing-laws-map/.22 (2017).

[21] Bhardwaj A. & Johri A. Don’t shoot the messenger. *The Hindu*. www.thehindu.com/opinion/op-ed/don’t-shoot-the-messenger/article19397939.ece/amp/ (2017).

[22] Blueprint for Free Speech. Whistleblowing laws gain traction across Africa. https://blueprintforfreespeech.net/whistleblwing-laws-gain-traction-across-africa/ (2017).

[23] Motarjemi Y. Whistleblowing: Food safety and fraud. *Food Safety Magazine* July 1–12 (2014).

[24] Soon JM, Manning L (2017). Whistleblowing as a countermeasure strategy against food crime. Br. Food J..

